# Strategies to Increase the Biological and Biotechnological Value of Polysaccharides from Agricultural Waste for Application in Healthy Nutrition

**DOI:** 10.3390/ijerph18115937

**Published:** 2021-06-01

**Authors:** María Ángeles Rivas, Rocío Casquete, Alberto Martín, María de Guía Córdoba, Emilio Aranda, María José Benito

**Affiliations:** 1 School of Agricultural Engineering, University of Extremadura, Avda. Adolfo Suárez s/n, 06007 Badajoz, Spain; mrivasm@unex.es (M.Á.R.); amartin@unex.es (A.M.); mdeguia@unex.es (M.d.G.C.); earanda@unex.es (E.A.); mjbenito@unex.es (M.J.B.); 2 University Institute of Agro-Food Resources Research (INURA), Campus Universitario, University of Extremadura, Avda. de la Investigación s/n, 06006 Badajoz, Spain

**Keywords:** agricultural waste, polysaccharides, bioactive function, extraction methods, nutraceuticals

## Abstract

Nowadays, there is a growing interest in the extraction and identification of new high added-value compounds from the agro-food industry that will valorize the great amount of by-products generated. Many of these bioactive compounds have shown beneficial effects for humans in terms of disease prevention, but they are also of great interest in the food industry due to their effect of extending the shelf life of foods by their well-known antioxidant and antimicrobial activity. For this reason, an additional research objective is to establish the best conditions for obtaining these compounds from complex by-product structures without altering their activity or even increasing it. This review highlights recent work on the identification and characterization of bioactive compounds from vegetable by-products, their functional activity, new methodologies for the extraction of bioactive compounds from vegetables, possibly increasing their biological activity, and the future of the global functional food and nutraceuticals market.

## 1. Polysaccharides from Agricultural Waste

Nowadays, 37 million tons of agricultural residues are generated worldwide [1] causing a serious economic and environmental problem [2]. These residues are made up of waste and plant by-products, such as skins and seeds. Plant by-products are rich sources of dietary fiber, soluble polysaccharides, phenolic compounds and fatty acids, making them particularly interesting for use as additives and functional ingredients.

Polysaccharides from agro-food industry waste constitute one of the most important renewable resources. The great variety of their chemical composition and structure, and their biodegradability and safety make them ideal for application in diverse fields, such as the food, pharmaceutical, cosmetics, tissue engineering and biofuels industries, among others [3,4].

### 1.1. Polysaccharide Classification

Natural polysaccharides from agricultural residues form part of the plant cell walls that are highly variable in terms of structure and composition [5]. In general, cell walls are composed of high molecular weight polysaccharides, which are mainly lignin, cellulose, hemicelluloses, pectins and other non-starch polysaccharides, such as inulin and oligosaccharides [6]. Polysaccharides that form cell walls are classified as insoluble and soluble based on their ability to be soluble in water. Insoluble polysaccharides are lignin, cellulose, hemicelluloses and pectins (insoluble); the soluble polysaccharide group consists of other pectins and hemicelluloses [7].

#### 1.1.1. Cellulose

Cellulose is the most abundant biopolymer on Earth, with an annual production of about 50 thousand tons [8]. Composed exclusively of β-glucose molecules linked by β-1,4-glycosidic bonds, cellulose is characterized by its capacity for chemical modification and hydrophilicity [9]. Cellulose is found in different agricultural residues, such as garlic skin [10], corn [11], grape pomace [12] and carrot [13]. Cellulose has multiple applications in the food industry; among others, its application as a fat substitute [14] has been proven to improve food texture [15], and it has also been widely used as a film to protect food. Riaz et al. [16] manufactured cellulose-based coatings from Chinese chive root extract, and the results showed that the coatings possessed antioxidant and antimicrobial properties.

#### 1.1.2. Hemicellulose

Hemicellulose is a heteropolysaccharide, composed of a heterogeneous set of polysaccharides, itself composed of two types of monosaccharides linked by β-bonds, which form a branched linear chain. It is the second most abundant component of agricultural residues, representing approximately 20–40% [17,18]. Hemicelluloses include glucans, xyloglucans, mannans, xylans and β-(1→3,1→4)-glucans [19]. However, the polysaccharides that constitute the majority of hemicelluloses are mannan and xylan [20].

Xylan is the most abundant hemicellulose found in nature [21]. Xylan from agricultural residues can be hydrolyzed and converted into xylose; furthermore, it can be turned into xylooligosaccharides (XOS) with different degrees of polymerization [22]. Aachary and Prapulla [23] reported that XOS with a degree of polymerization of 2–3 have maximum prebiotic potential. XOS are extracted industrially from corn and sugarcane [24,25], although they can also be obtained from agricultural by-products such as pineapple rind [26], straw rice [27] and quinoa stems [28].

Another polysaccharide that is part of the hemicelluloses is mannan, which is significantly present in agricultural residues. By enzymatic hydrolysis, mannan can be turned into mannooligosaccharides (MOS) [29]. MOS are considered as emerging prebiotics and can be obtained from different agricultural residues, such as copra flour [30].

#### 1.1.3. Pectic Polysaccharides

Pectins, mostly considered soluble fiber, are part of the cell wall of plants and are heteropolysaccharide polymers rich in polygalacturonic acid that can be composed of up to 17 different monosaccharides [31], which is why it is characterized as one of the most structurally complex natural plant polysaccharides [32]. It is composed of three structurally distinct domains: homogalacturonan (HG), rhamnogalacturonan (RG-I) and rhamnogalacturonan (RG-II).

Pectins are traditionally obtained from agricultural by-products, such as citrus peels and apple pomace [33,34]. The increasing global demand for this heteropolysaccharide due to the numerous health benefits attributed to it [35] means that alternative sources of pectin are being sought in other vegetables and by-products, such as eggplant [36], tomato peel [37,38], broccoli stem [39] and pomegranate peel [40], among others.

The main component of pectins is HG, a polymer with a linear homogeneous chain of α-1,4-glycoside linked to D-galacturonic acid [41]. Methoxy esters, located in the C6 carboxyl groups of D-galacturonic acid, are substitutions generally found in the HG region and play an important role in the known functional properties and health benefits of these pectic polysaccharides [42]. RG-I is the second most abundant pectic polysaccharide in the cell wall of plants [43]. It is composed of a repeating backbone of galacturonic acid and rhamnose disaccharide, usually with neutral side chains [44]. RG-I polysaccharides have been demonstrated to have a number of promising bioactive properties [45]; their bioactivity is attributed to their composition and structure [46]. Although less common in the pectic fraction, RG-IIs are polysaccharides with abundant bioactive properties and many human health benefits. Their structure comprises a main chain linked to galacturonic acid and side chains of highly complex oligosaccharides and other unusual monosaccharides [47].

In addition, depolymerization of pectin releases pectic oligosaccharides (POS) [48]. POS are currently described as emerging prebiotics with numerous health benefits [49].

### 1.2. Bioactive Function

Table 1 summarizes the applications of polysaccharides obtained from by-products in the food industry. In addition to the prebiotic effect, polysaccharides have been studied for their antioxidant and antimicrobial activity; due to this activity, important effects have been reported: the prevention of diseases such as cancer, the regulation of chronic metabolic syndrome, application as immunomodulators and anti-inflammatories, and anti-diabetic, anticoagulant and antiviral activity. Additionally, technological properties are found, since they are used as a fat substitute, to improve the texture of food and as a meat preservative.

#### 1.2.1. Prebiotic Activity

Significant research indicates that polysaccharides derived from plant cell walls have high prebiotic activity [50] and are able to stimulate the growth of certain bacteria and promote the production of certain short-chain fatty acids (SCFA) [51].

Many authors have demonstrated the prebiotic effect of different polysaccharides from agricultural waste; in vitro experiments performed with XOS extracted from barley straw demonstrated their suitability for use as a prebiotic ingredient [52]. Another type of hemicellulosic polysaccharide that demonstrates prebiotic character is MOS as they resist digestion in the upper gastrointestinal tract [53]. Pectic polysaccharides have also shown prebiotic activity. The prebiotic properties of RG-I have been tested; Khodaei et al. [54] extracted RG-I from potatoes and demonstrated the growth of beneficial bacteria (*Bifidobacterium* spp. and *Lactobacillus* spp.). POS were extracted from sugar beets, and selective growth was observed of bacteria of the genus *Lactobacillus* [55].

#### 1.2.2. Antioxidant Activity

Nowadays, there is growing evidence that many types of polysaccharides possess important antioxidant activity. Zeng et al. [56] investigated the structural characteristics of two polysaccharides obtained from Chinese water chestnut shells and showed that both had potential antioxidant activity. The low molecular weight polysaccharide extracted from *Cucurbita moschata* also showed proven antioxidant activity, exhibiting significant scavenging ability against ABTS and DPPH radicals [57]. Heteropolysaccharides composed of rhamnose, glucose, galactose, mannose, xylose, arabinose and galacturonic acid extracted from pistachio husk showed significant antioxidant potential [58]. Four pectin-like polysaccharides (PKP-E-1− 1, -1− 2, -2− 1, and -2− 2) extracted from *Pinus koraiensis* pineapples showed promising potential antioxidant properties for food applications [59]. The antioxidant activity was measured by determining the elimination capacity of hydroxyl radicals of polysaccharides and comparing it with that of ascorbic acid.

Oxidative stress caused by free radicals has been involved in the pathogenesis of several human diseases, as natural antioxidant defenses have been found to be defective in some of the same diseases. This has led to the suggestion that disease progression may be slowed by supplementation with natural antioxidants. Antioxidant therapy may be beneficial in diseases including diabetes mellitus, reperfusion injury and inflammatory diseases, as well as in the prevention of chronic processes such as atherosclerosis and carcinogenesis.

**Table 1:** Summary of applications of polysaccharides from by-products in the food industry.

Compound	By-Products	Beneficial Effects	References
Cellulose	Garlic skin, corn, grape pomace, carrot, Chinese chive root extract	Technological processes: as a fat substitute, to improve the texture of food. Nutritional, antioxidant and antimicrobial activity.	[10,11,12,13,14,15,16]
Xylan/xylooligosaccharides, methylglucuronoxylan (hemicellulose)	Corn (corncobs), sugar cane, pineapple rind, straw rice, quinoa stems, soybean, mango seed, barley straw, neem plant, Spanish chestnut	Prebiotic activity Disease prevention: preventing cancer	[22,23,24,26,27,28,52,60,61]
Mannan/mannooligosaccharides (hemicellulose)	Copra flour/meal, palm kernel cake, guar gum, potato peel	Prebiotic activity	[29,30]
Pectins, pectic oligosaccharides (POS), homogalacturonan (HG), rhamnogalacturonan (RG-I), rhamnogalacturonan (RG-II)	Citrus peel, mandarin citrus peel, apple pomace, eggplant, tomato peel, broccoli stem, pomegranate peel, potato pulp	Prebiotic activity Disease prevention: preventing cancer, regulating chronic metabolic syndrome, immunomodulatory, anti-inflammatory and probiotic uses	[33,34,36,37,38,39,40,54,55,62,63,64,65,66,67,68,69,70]
Heteropolysaccharides, WVP-1 (mannose, glucose, galactose and arabinose) WVP-2 (mannose, rhamnose, glucuronic acid, galacturonic acid, glucose, galactose and arabinose)	Mango pomace, chestnut shells	Disease prevention: preventing cancer, immunomodulatory, anti-diabetic, anticoagulant, antiviral Biological activity: antioxidant activity	[56,71]
Natural low molecular weight polysaccharides (SLWPP-3)	Pumpkin by-products	Biological activity: antioxidant activity Disease prevention: anti-diabetic	[72,73]
Heteropolysaccharides (rhamnose, glucose, galactose, mannose, xylose, arabinose and galacturonic acid)	Pistachio external hull	Technological process: meat preservative Biological activity: antioxidant activity	[58]
Heteropolysaccharides: PKP-E	Pinecones	Biological activity: antioxidant activity	[59]
Glucan, inulin	Cereals	Biological activity: immunomodulatory, anti-inflammatory, antimicrobial activity	[74,75,76]

#### 1.2.3. Anti-Diabetic Activity

Diabetes is a growing global problem and a heavy financial cost burden on health services. Millions of people suffer from the disease which causes many deaths each year, as well as being associated with an increased risk of other health problems. Polysaccharides have a significant hypoglycemic effect and therefore may be useful to prevent diabetes mellitus resulting from defects in insulin production or action that cause hyperglycemia. Li et al. [72] reported the hypoglycemic effects of polysaccharides extracted from pumpkin by-products. Different by-product polysaccharides have gained popularity among researchers due to their numerous bioactive properties, including inhibitory effects against starch hydrolyzing enzymes such as α-amylase and α-glucosidase, highlighting their potential as anti-diabetic agents in the treatment and prevention of diabetes mellitus [73].

#### 1.2.4. Anticancer Activity

The anticancer effect of natural polysaccharides from plant residues is currently under study. Sharma et al. [61] demonstrated in their study that XOS extracted from sawdust of *Azadirachta* inhibits the growth of human colorectal cancer cells (HT-29). In another study, three major polysaccharides were isolated from mango pomace, composed of seven monosaccharides (mannose, rhamnose, glucose, galactose, xylose, arabinose and fucose) and two uronic acids, and the isolated polysaccharides showed significant anticancer activity against HepG2, MCF-7, A549, HeLa, A2780, HCT-116 and BGC- cells [71]. HG from the extraction of *Hippophae rhamnoides* berries has also been shown to have an antitumor effect [64]. RG-I have been shown to be able to promote cell adhesion and migration [77] and immunomodulatory activity [62].

#### 1.2.5. Anti-Inflammatory Activity

Natural polysaccharides such as glucan [74], inulin [75] and pectins [78] have been shown to have strong anti-inflammatory activity. As demonstrated by Bermudez-Brito et al. [63], pectic polysaccharides exhibit higher anti-inflammatory activity than inulin and glucan. Moreover, pectic polysaccharides show high anti-inflammatory activity in their three domains (HG, RG-I and RG-II) [70].

Hosary et al. [76] isolated glucan from the plant *Avena sativa* L. and demonstrated its anti-inflammatory capacity, showing its high potential for use as a wound-healing hydrogel; it also showed antimicrobial activity against *Staphylococcus aureus* and *Micrococcus luteus*.

#### 1.2.6. Antimicrobial Activity

Several studies have shown the antimicrobial activity of natural polysaccharides as they have a strong ability to inhibit the growth of a wide range of infectious and spoilage micro-organisms. Rostami et al. [79] extracted polysaccharides from *Malva sylvestris* and showed that Gram-positive bacteria (*Bacillus cereus* PTCC 1015 and *Staphylococcus aureus* PTCC 1112) were less sensitive than Gram-negative bacteria (*Escherichia coli* PTCC 1763 and *Salmonella typhimurium* PTCC 1709) to the different polysaccharide extracts obtained. However, Hosary et al. [76] developed a hydrogel using polysaccharides derived from Egyptian *Avena sativa* L. The developed product showed slight activity against *Candida albicans* and high activity only against the Gram-positive bacterial strains *Staphylococcus aureus* ATCC 297373 and *Micrococcus luteus* ATCC 10240, and none against the two Gram-negative strains used in the study, *Escherichia coli* ATCC 10536 and *Pseudomonas aeruginosa* ATCC 25619. In the same way, Hashemifesharaki et al. [80] obtained polysaccharides by microwave-assisted extraction of marshmallow root and increased the antimicrobial activity of the polysaccharide extract mainly against Gram-positive bacteria, in particular, *Staphylococcus aureus* PTCC 1189 and *Bacillus circulans* ATCC 4516.

## 2. Extraction of Polysaccharides: Methods and Influence on the Bioactive Function

The different extraction methods, the extraction solvent, the pH, the ratio of raw material to solvent, the temperature and the time have a significant influence on the yield, technological properties and functions of bioactive polysaccharides [81,82]. Each extraction method has its advantages and disadvantages; therefore, the extraction method chosen should be adapted to the final purpose, the nature of the by-product and the cost of the procedure.

Table 2 shows an overview of the optimized extraction methods and their influence on the bioactive function of polysaccharides obtained from plant by-products.

### 2.1. Hot Water Extraction (HWE)

HWE is one of the most widely used methods to extract polysaccharides, being conventional, simple and cheap. However, the use of HWE is limited due to its low yield; only extracellular polysaccharides can be obtained since the cell wall is not degraded [83]. High temperatures and long extraction times are needed to achieve high yields [84], which results in degradation of the structure and a decrease in quality and bioactivity [85,86]. Therefore, there is a need to explore new methods of polysaccharide extraction that ensure a good yield besides maintaining the bioactive and functional characteristics of the polysaccharides.

Several authors have reported the optimization of HWE conditions and assurance of good yields. Romdhane et al. [87] have reported the effect of temperature, time and ratio of water to raw mate-rial on the extraction yield of WMRP using the classical “one factor at a time” methodology. These authors obtained a good yield of polysaccharides extracted from watermelon rind, and they showed good functional activity and significant antioxidant capacity. Khatib et al. [88] showed that the use of hot water maximized solubility and extractability of the crude polysaccharides from Laffan and Wonderful pomegranate mesocarp with prebiotic properties in vitro by serving as an excellent substrate for the growth of potentially probiotic bacteria, such as *Lactobacillus* and *Bifidobacterium* strains. Sharifian-Nejad et al. [89] optimized the extraction of polysaccharides from oleaster fruits depended on temperature, water/dry matter ratio, time, and alcohol ratio and the highest polysaccharide purity at a temperature of 60 °C; and obtained the best results at 53:1 water/dry matter ratio (*V*/*W*); time, 5 h; and alcohol ratio of 2.9 (*V*/*V*) with a solubility of 67.46%.

### 2.2. Ultrasound-Assisted Extraction (UAE)

UAE is based on a phenomenon called acoustic cavitation, which involves the generation and formation of gas vapor-filled bubbles in a liquid that expand and finally collapse. Cavitation generates circulating liquid currents and turbulence as well as an increase in temperature and pressure [90]. This leads to an increase in the overall extraction yield [91]. Among the advantages of UAE are that it is considered one of the most cost-effective techniques for the extraction of polysaccharides [90], apart from being efficient, fast and environmentally friendly.

UAE has been used to extract pectic polysaccharides from the peel of fruit and vegetables, such as eggplant [36], pomegranate [92], tomato [93], custard apple [94] and mango [95]. UAE of tomato peel was able to efficiently extract two valuable bioactive ingredients (pectin and polyphenols) simultaneously, in addition to shortening the extraction time with respect to conventional extraction techniques [37]. It has also been reported that high yields of hemicellulose polysaccharides can be obtained with short extraction times, especially xyloglycans by UAE in grape pomace [96]. UAE turns out to be efficient for extracting prebiotic sugars from industrial artichoke residues: 1-kestose, nystose, fructofuranosylnystose and raffinose were successfully extracted, obtaining an extract of approximately 9.6 mg of prebiotic saccharides/g of dry raw material [97].

### 2.3. Microwave-Assisted Extraction (MAE)

MAE involves the penetration of electromagnetic radiation into a solid matrix. The heating generated is due to the molecular friction caused by the ionic conduction of the dissolved ions and the rotation of the dipoles of the polar solvent, which favor the extraction of the bioactive compounds. Both the heating produced and the internal pressure originated cause rupture of the cell; as a consequence, the structure is altered, which facilitates the release into the solvent of the bioactive compounds, improving the transfer coefficient [98,99,100]. MAE is a promising technique for polysaccharide extraction; it has advantages such as high yields, less solvent used, shorter extraction times and being environmentally friendly [101,102,103].

Currently, MAE is widely used in the extraction of polysaccharides from various sources of by-products, such as banana peel [104], grapefruit peel [105], broccoli by-products [106] and cocoa shell [107], among others. Dao et al. [108] reported higher yields (18.73%) and higher purity of pectic polysaccharides extracted from fruit peels, such as dragon fruit and passion fruit, compared to conventional methods. In addition, extraction times were shortened, resulting in a reduction in energy consumption. High yields of hemicellulose polysaccharides such as xylan, glucuronoxylan and xyloxylan extracted by MAE from tobacco plant residues have also been reported [109].

**Table 2:** Review of optimized extraction methods and their influence on the bioactive function of polysaccharides obtained from plant by-products.

Optimized Extraction Method	Compounds	By-Products	Influence on Bioactive Function	Reference
Hot water extraction	Polysaccharides	White mulberry	Anti-diabetic, immunomodulatory, anti-inflammatory, antioxidant, hepatoprotective, renoprotective and anti-obesity activity; effect on gut microbiota	[84]
Hot water extraction	Polysaccharides	Watermelon rind	Antihypertensive and antioxidant activity	[87]
Hot water extraction	Polysaccharides	Pomegranate fruit	Prebiotic activity	[88]
Hot water extraction	Polysaccharides	Oleaster fruit	N.d. *	[89]
Ultrasound-assisted extraction	Polysaccharides/starch, pectin	Yam tubers, fruit peel, tomato processing, potato…	Antioxidant, anticoagulant, antitumor, anti-inflammatory and prebiotic activity	[90]
Ultrasound-assisted extraction	Polysaccharides/pectin	Fruit and vegetable peel: eggplant	Antioxidant activity	[36]
Ultrasound-assisted extraction	Polysaccharides/pectin	Pomegranate peel	N.d. *	[92]
Ultrasound-assisted microwave extraction	Polysaccharides/pectin	Tomato peel	N.d. *	[93]
Ultrasound-assisted extraction	Polysaccharides/pectin	Custard apple peel	N.d.*	[94]
Ultrasound-assisted extraction	Polysaccharides/pectin	Mango peel	N.d. *	[95]
High hydrostatic pressure and ultrasound-assisted extraction	Polysaccharides/pectin	Tomato peel	N.d. *	[37]
Ultrasound-assisted extraction	Hemicellulose polysaccharides/xyloglycans	Grape pomace	N.d. *	[96]
Ultrasound-assisted extraction	Fructooligosaccharides	Artichoke industrial waste	Prebiotic activity	[97]
Microwave-assisted extraction	Polysaccharides	Marshmallow roots	Antioxidant, antimicrobial and antitumor activity	[80]
Microwave-assisted extraction	Polysaccharides	*Chuanminshen violaceum* root	Increased antioxidant activity	[86]
Microwave-assisted extraction	Polysaccharides/pectin	*Carica papaya* L. peel	N.d. *	[98]
Microwave-assisted extraction	Polysaccharides	Kiwifruit	Antioxidant activity	[99]
Microwave-assisted extraction	Polysaccharides	*Camptotheca acuminata* fruits	Antioxidant and immunomodulatory activity	[100]
Surfactant and microwave-assisted extraction	Polysaccharides/pectin	Orange peel	N.d. *	[101]
Microwave-assisted extraction	Polysaccharides	Waste jamun fruit seeds	N.d. *	[102]
Microwave-assisted extraction	Polysaccharides	*Sargassum pallidum*	Hypoglycemic activity	[103]
Microwave-assisted extraction	Polysaccharides/pectin	Banana peel	N.d. *	[104]
Hot-solvent microwave extraction	Polysaccharides/pectin	Pomelo peel	N.d. *	[105]
Microwave hydrodiffusion and gravity	Polysaccharides	Broccoli	N.d. *	[106]
Microwave-assisted extraction	Polysaccharides	Cocoa bean shell	Antioxidant activity	[107]
Microwave-assisted extraction	Polysaccharides/pectin	Fruit peels	N.d. *	[108]
Microwave-assisted extraction	Hemicellulose polysaccharides/xyloglycans	Tobacco plant residues	N.d. *	[109]
Enzyme-assisted extraction	Polysaccharides	*Fritillaria pallidiflora* Schrenk	Antioxidant, antimicrobial, anti-inflammatory, antitumor and antihypertensive activity	[110]
Enzyme-assisted extraction	Polysaccharides	*Malva sylvestris* plant	Increased antioxidant, antitumor and antimicrobial activity	[79]
Enzyme-assisted extraction	Polysaccharides	Cup plant (*Silphium perfoliatum* L.)	Antioxidant and hypoglycemic activity	[111]
Enzyme-assisted extraction	Polysaccharides/pectin	Kiwi pomace	N.d. *	[112]
Enzyme-assisted extraction	Polysaccharides/pectin	Apple pomace	Antioxidant and anticancer activity	[113]
Enzyme-assisted extraction	Polysaccharides/pectin	Pomegranate peel	Antioxidant activity	[114]
Enzyme-assisted extraction	Polysaccharides	*Dendrobium chrysotoxum*	Immunological activity	[115]
Enzyme-assisted supercritical fluid extraction	Polysaccharides	Pomegranate peel	Antioxidant activity	[116]
Supercritical fluid extraction	Polysaccharides	*Artemisia sphaerocephala* Krasch. seeds	N.d.*	[117,118]
Supercritical fluid extraction	Polysaccharides	Pomegranate peel	Antioxidant activity	[118]
Deep extraction with eutectic solvent/microwave-assisted extraction	Polysaccharides	Bladder-wrack (*Fucus vesiculosus*)	Antioxidant activity, cell growth inhibition	[119]
Accelerated solvent extraction	Polysaccharides	Bamboo shoots	Antioxidant activity	[120]
Dynamic high-pressure microfluidization	Polysaccharides	*Nelumbo nucifera* leaves	Antioxidant activity	[121]
Ultrasonic-cellulase synergistic extraction	Polysaccharides	Pineapple pomace	Hypoglycemic and anticancer activity	[122]
Deep extraction with eutectic solvent	Polysaccharides/pectin	Pomelo peel	N.d. *	[123]

* Not determined.

### 2.4. Enzyme-Assisted Extraction (EAE)

While the UAE and MAE methods break the cell wall, EAE degrades the cell parts by enzymatic hydrolysis, which causes an improvement in the yield and biological activity of polysaccharides [110,124]. The method is selective for the extracted bioactive compounds and environmentally friendly [80]. The extraction yield depends on several factors, such as the liquid–solid ratio, pH, amount of enzyme, temperature and extraction time [111,125].

EAE has been used effectively for extraction of pectin from kiwifruit pomace, demonstrating a higher pectin yield by enzymatic extraction with Celluclast (cellulases, polygalacturonase, pectin lyase and rhamnogalacturonan lyase) than by acid extraction with citric acid [112]. Wikiera et al. [113] also reported higher extraction yields for pectin (15.3%) from apple pomace by EAE compared to acid extraction with sulfuric acid. EAE achieved higher polysaccharide extraction yields from pomegranate peel than those obtained with HWE and UAE, the extracted polysaccharides having strong antioxidant properties [114]. Extraction of polysaccharides from *Dendrobium chrysotoxum* by EAE provided 1.25-fold higher yields than with HWE [115].

### 2.5. Supercritical Fluid Extraction (SFE)

SFE is an emerging technology in the extraction of bioactive compounds that allows the natural qualities of the compounds to be preserved and ensures food safety [126]. The critical point of CO_2_ (31 °C and 7.38 MPa) allows the recovery of bioactive compounds with a high degree of purity and especially useful clean extracts for functional foods [116]. SFE technology has, to a large extent, been used for apolar substances, although selective extraction of polar compounds is possible by using modifications. Therefore, the use of cosolvents, such as ethanol and methanol, can increase the efficiency of polysaccharide extraction [118]. However, SFE conditions and information available on polysaccharide extraction and its properties are limited. Chen et al. [117] reported a yield of 18.59% for SFE of polysaccharides from *Artemisia sphaerocephala* plants with extraction parameters of 45 MPa at 45 °C, with a CO_2_ flow of 20 L/h for 2 h. Rivas et al. [118] optimized SFE using CO_2_ to obtain high-value compounds from pomegranate peel by-products.

### 2.6. Other Extraction Methods

New polysaccharide extraction methods are being developed, such as deep extraction with eutectic solvents [119], accelerated extraction with solvents [120,123] and high-pressure dynamic microfluidization [121]. In addition, the previously mentioned methods are also combined to improve the yields and bioactive functions of polysaccharides.

The polysaccharides, mannose, rhamnose, glucose, galactose, xylose, arabinose, fucose, glucuronic acid and galacturonic acid, were extracted from pineapple pomace by combining UAE and EAE to provide ultrasonic-cellulase synergistic extraction; the extracted polysaccharides demonstrated the ability to inhibit development of HepG2 cells resistant to insulin and therefore can be considered potential ingredients to develop a new beneficial food [122]. In addition, Liew et al. [123] reported a 1.5 to 3.5 times higher yield for the extraction of pectic polysaccharides combining UAE and EAE compared to separate extraction methods.

## 3. Strategies to Increase the Biological Activity

The bioactivity of polysaccharides depends on their structure, monosaccharide units, bond type, spatial configuration, branched-chain distribution and molecular weight [127,128]. The high molecular weight of polysaccharides in nature makes it necessary to modify and degrade polysaccharides to improve their solubility and make them more accessible to cells [129,130]. Chemical [131], physical [80] and biological [57,132] methods are used to modify and degrade polysaccharides, thereby reducing their size and molecular weight to improve their bioactivity.

### 3.1. Enzymatic Modification

Enzymatic degradation improves the properties and bioactivity of polysaccharides by modifying their structure and reducing the molecular weight [133]. The main advantage is the high selectivity, which allows polysaccharides to be modified in a personalized way, obtaining well-defined structures [134]. Enzymatic modification, in addition to improving polysaccharide properties and oligosaccharide production [133], also increases the possibility of extracting non-extractable polyphenols associated with cell wall polysaccharides [135,136,137].

The acquisition of POS with a specific degree of polymerization (DP) by enzymatic treatment of pectic polysaccharides extracted from onion skins has been reported. Pectin treatment was carried out with three enzymes (EPG-M2, Viscozyme and pectinase), EPG-M2 being the one that showed the best results for the production of POS [138]. Mathew et al. [139] improved by means of enzymatic treatment the soluble polysaccharide arabinoxylan extracted from wheat bran through the action of enzymes (endoxylanases GH10 and GH11), managing to produce XOS and arabino-xylooligosaccharides with prebiotic potential. Enzyme treatment with α-amylase and amyloglucosidase of the immunostimulatory polysaccharides isolated from red ginseng was proven to improve the modulating activity of the intestinal immune system compared to non-enzymatically treated polysaccharides [140].

### 3.2. Ultrasound Modification

Ultrasound degradation is a physical method that allows operation at high or low frequency depending on the intended purpose [90]. Degradation by ultrasound modifies the molecular weight and solubility and improves the properties and bioactivity of polysaccharides [141,142]. In addition, ultrasound treatment is an environmentally friendly, fast method that saves energy [143].

Chen et al. [144] demonstrated in their study the effect of ultrasound treatment on the digestibility of five fractions of polysaccharides extracted from bamboo shoots (*Chimonobambusa quadrangularis*). The results indicated that there was an improvement in the prebiotic potential of the polysaccharides. Similarly, Zeaiter et al. [145] reported higher prebiotic activity in artichoke polysaccharides extracted using the ultrasound technique compared to other treatments. In addition to improving prebiotic activity, modification by ultrasound has been shown to enhance other bioactive properties, such as antioxidant activity, ultrasonic treatment being reported to improve the antioxidant activity of pectic polysaccharides extracted from hawthorn [146]. Modification by ultrasound has also been reported to improve the bioactivity of polysaccharides, such as anti-inflammatory [147] and antitumor properties [148].

### 3.3. Microwave Modification

The treatment of polysaccharides by microwave technology is a promising way to obtain polysaccharides with suitable properties and bioactivity. The bioactivity of polysaccharides of natural origin is conditioned by their structure, composition and number of monosaccharides, molecular weight, type of bond and DP [149]. Therefore, despite being a promising approach, it is a novel technique for polysaccharide improvement, and the effect on bioactivity needs to be further investigated and studied. However, there is evidence that polysaccharides obtained and treated with microwave technology have antioxidant [150], antifungal [151], antiviral [152], antitumor [80] and antibacterial activity [153].

The minimum inhibitory concentrations of the pathogens *E. coli*, *B. subtilis* and *S. aureus* were determined in the presence of microwave-treated and conventionally treated ginseng polysaccharides, and higher antibacterial activity was reported for microwave-treated polysaccharides compared to those that were treated with conventional technology [6]. Hashemifesharaki et al. [80] demonstrated in their study that microwave treatment is able to purify the homogeneous fractions of polysaccharides extracted from marshmallow root, and these showed significant improvement in their antiradical, antioxidant, antimicrobial and antitumor activity.

### 3.4. Other Treatments

Other techniques are being investigated for the modification of polysaccharides, such as cold plasma processing [154,155], pulsed electric field processing [156], high pressure [83], high-pressure UAE [157,158], supercritical fluids [159,160] and degradation with ascorbic acid [161,162].

Cold plasma is an emerging technology in the processing of temperature-sensitive biological compounds [163], and it is also considered an environmentally friendly technology [164]. It has been used to modify the properties and bioactivity of polysaccharides [165]. The polysaccharide galactomannan was extracted from the seeds of the legume fenugreek and modified by cold plasma degradation; the results showed improved functional and rheological properties of the polysaccharide [166].

Pulsed electric field technology is an environmentally friendly non-thermal technology [167]. It is used for the extraction and modification of polysaccharides and oligosaccharides, such as pectin [168,169,170] and inulin [171], improving their properties.

High pressure is an increasingly popular non-thermal method in the processing of bioactive compounds. It has been used for the modification of macromolecules, significantly improving their functional properties and bioactivity. It acts on polysaccharides by causing changes in their structure, bonding, and monosaccharide composition and number [172]. Porfiri et al. [173] showed the high potential of high pressure to modify polysaccharides, achieving the solubilization of certain compounds in soybean regarded as insoluble polysaccharides.

## 4. Future Perspective: Nutritional Therapy

**Figure 1:**
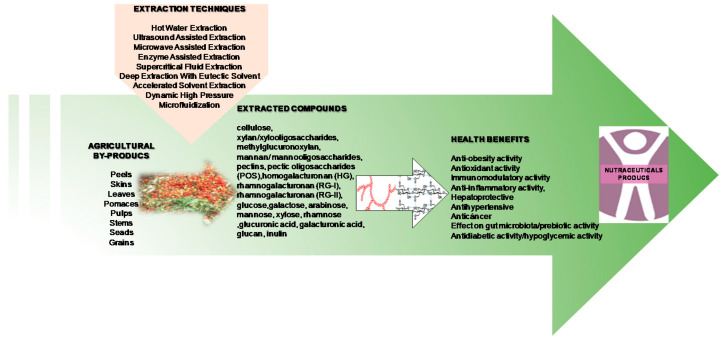
Overview of extraction of agricultural waste by-product polysaccharides with different health-beneficial activity for nutraceutical use.

Polysaccharides obtained from agricultural waste by-products are heterogeneous and structurally different, which complicates the research. The beneficial effects of these polysaccharides have already been demonstrated, and they could be used as nutraceuticals. Nutraceuticals, according to the recognized definition, are foods or parts of foods that confer medical or health benefits, including the prevention and/or treatment of a disease. The term “nutraceutical” was introduced in 1989 by Stephen DeFelice and combines two words: “nutrient” (a nutritional food component) and “pharmaceutical” (a drug) [174]. Polysaccharides obtained from agricultural waste usually have limited aqueous solubility and bioavailability that can be improved by using the technologies mentioned earlier in this review. Figure 1 shows a summary of the different polysaccharides obtained from agricultural waste, their health benefit applications and methods for obtaining them.

It is of great interest to explore the therapeutic use of these polysaccharides in humans, considering their prebiotic, antioxidant, anticancer, hypoglycemic, anti-inflammatory and antimicrobial activity. Current therapeutic strategies are focused on the use of natural compounds to avoid the use of drugs that are often ineffective and have adverse health effects. Although more studies should be developed in this field, recently, several different non-digestible polysaccharides have been studied for their therapeutic use, most of them focused on intestinal health and intestinal microbiota [175]. Different seaweed polysaccharides, used for drug development, have been shown to be effective for the treatment of Inflammatory Diseases of the Intestine [176]. Saeed et al., [177] described the therapeutic benefits of a fucose-rich polysaccharide from brown algae, defining its importance for the treatment of ischemic diseases and as an anticancer agent to treat colon cancer. Other medicinal plants, which are potential sources of biologically active polysaccharides, showed immunological, antitumor, antihyperlipidemic, cardioprotective, analgesic, anti-inflammatory, antidiabetic, antioxidant and hepatoprotective effects. Several polysaccharides isolated from different parts of *Lagenaria siceraria* medicinal plant [178] have been proposed for therapeutic use.

## 5. Conclusions

In order to obtain the best characteristics of the polysaccharides for therapeutic use, researchers have observed how the method used to acquire these compounds can be crucial for increasing the concentration of the polysaccharides extracted and significantly improving their biological activity. The methods reviewed here have been optimized to enhance the solubility rate and decrease the molecular size of polysaccharides, improving their bioavailability without modifying their healthy functional properties. According to the existing studies and literature resources, careful attention should be focused on the bioactive effect of polysaccharides from agricultural waste under in vivo clinical conditions. The use of these functional polysaccharides will be a promising approach in promoting public health. Further research is needed to elucidate the relationship between chemical structure, biological activity and behavior at the gastrointestinal tract level.
